# Supplementary material for: WordCommentsAnalyzer: A windows software tool for qualitative research
Source: F1000Res. 2018 Sep 4;7:536. Originally published 2018 May 3. [Version 2] doi: 10.12688/f1000research.14819.2 (PMC6137411; doi:10.12688/f1000research.14819.2)
Supplement: Supplementary file 3 [file f1000research-7-17614-s0002.tgz › b2a79496-4ada-47c4-9682-a99ce867836d.zip!/Eight Iranian Health Journals/Salmand_Iranian Journal of Ageing _27.docx]

© Salmand_Iranian Journal of Ageing . This work is licensed under CC BY-NC 4.0 (https://creativecommons.org/licenses/by-nc/4.0/).

Volume & Issue: دوره 6 - شماره 3

Title: بررسی تأثیر بازآموزی تعادل چند حسی بر کنترل وضعیتی سالمندان

http://salmandj.uswr.ac.ir/article-1-443-fa.html

اهداف: افراد سالمند به صورت چشمگیری بیشتر از افراد جوان در شرایطی که تداخل حسی به وجود می آید دچار نوسان می شوند و احتمال افتادن بیشتری دارند. با توجه به چشم انداز افزایش جمعیت سالمندی در ایران آینده و عدم کنترل پوسچر مناسب و بالا بودن هزینه های عواقب احتمالی آن دراین پژوهش به بررسی اثر آموزش تعادل چند حسی بر کنترل وضعیتی سالمندان و مقایسه آن با گروه کنترل پرداخته شده است. مواد و روش‌ها: در این مطالعه نیمه تجربی از روش نمونه گیری غیر احتمالی ساده استفاده شد و 34 زن سالمند سالم در دو گروه مداخله (19) و کنترل (15) به ترتیب با میانگین سنی 72/9و 72/4 سال در مطالعه شرکت نمودند. قبل و بعد از مداخلات درمانی جهت بررسی میزان کنترل پوسچرال و تعادل عملکردی سالمندان به ترتیب از صفحه نیرو و تست برگ استفاده شد.گروه مداخله در برنامه تعادلی چند حسی به مدت 5 هفته و هفته ایی 3 جلسه 1 ساعته شرکت کردند.تجزیه و تحلیل داده ها با استفاده از آزمونهای آماری تی مستقل و تی وابسته انجام شد. یافته‌ها: نتایج آزمون تی مستقل نشان داد بعد از طی دوره مداخله، تغییرات کنترل پوسچرال در دو شاخص طول مسیر و سرعت میانگین در حالت چشم باز (0/001=P) و چشم بسته (0/0001=P) و تست برگ (0/002=P) بین گروه مداخله و کنترل تفاوت معنی داری وجود دارد. نتیجه‌گیری: پژوهش حاضر نشان می دهد که تمرینات تعادلی چند حسی حتی در مدت زمان کوتاه می تواند باعث بهبود کنترل پوسچرال و همچنین تعادل عملکردی سالمندان شود.

Title: تأثیر مصرف چای بر خطر شکستگی ناشی از پوکی استخوان در سالمندی: متاآنالیز مطالعات مشاهده‌ای

http://salmandj.uswr.ac.ir/article-1-496-fa.html

اهداف: در مورد نقش چای در پوکی استخوان مطالعات متعددی منتشر شده است که در بسیاری از آنها نتایج ضد و نقیضی گزارش شده است. این متاآنالیز با هدف بررسی تأثیر مصرف چای بر روی خطر شکستگی استخوان انجام شده است. مواد و روش‌ها: این مطالعه بصورت مرور سیستماتیک و متاآنالیز بر روی مقالات منتشر شده بین سالهای 1980 تا 2010 انجام شد. به این منظور ابتدا بر اساس جستجو در پایگاه های اطلاعات پزشکی MEDLINE ،Pubmed ،ISI ،EMBASE و Cochrane با استفاده از یک استراتژی جستجوی مقالات و با کلمات کلیدی به‌دست آمده از MESH شامل Caffeine و Tea و Coffee و مشتقات آنها و بررسی ارتباط آنها با کلمات کلیدی Osteoporosis ،Bone density ،BMD (Bone Mineral Density) و Fracture، کلیه مقالاتی که از سال 1980 تا کنون در این زمینه به زبان انگلیسی منتشر شده‌اند، توسط دو پژوهشگر مستقل استخراج شدند. سپس با مطالعه خلاصه مقالات، مطالعاتی را که کاملا بی‌ارتباط با سوال تحقیق بوده، حذف شده و با بررسی متن کامل مقالات، مقالات کاملا مرتبط با هدف این پژوهش، انتخاب شدند. در مرحله آخر از میان مقالات انتخاب شده بر اساس معیارهای ارزشیابی نقادانه، مقالاتی که شرایط ورود به مطالعه را داشتند جهت آنالیز انتخاب گردیدند. یافته‌ها: در جستجوی اولیه، تعداد 421 مقاله استخراج شد که بعد از بررسی عناوین و خلاصه مقالات، 78 مقاله برگزیده شده و متن کامل آن‌ها تهیه و بررسی شد که از میان آنها تنها 8 مقاله شرایط لازم برای شرکت در آنالیز را داشتتند. متاآنالیز انجام شده بر روی این مطالعات مشاهده‌ای که تاثیر مصرف چای بر روی شکستگی استخوان گردن فمور را گزارش کرده بودند نشان می‌دهد که مصرف چای اثر پیشگیرانه در بروز شکستگی دارد ولی این تاثیر معنی‌دار نیست (RR=0/872، 0/733-1/038). در صورتیکه آنالیز را به تفکیک نوع مطالعه انجام دهیم مشاهده می شود که بر اساس نتایج منتشر شده در مطالعات کوهورت، مصرف چای به‌صورت معنی‌داری در کاهش خطر شکستگی استخوان گردن فمور موثر بوده است (RR=0/749، 0/603-0/929) ولی مطالعات مورد شاهدی افزایش مختصری در این احتمال خطر را نشان داده اند که از لحاظ آماری معنی‌دار نیست (RR=1/157، 0/863-1/553). نتیجه‌گیری: چای به عنوان نوشیدنی رایج، علاوه بر اثرات آنتی‌اکسیدانی فراوان ناشی از دارا بودن مقادیر بالای فلاونوئیند‌ها و پلی‌فنول‌ها، می‌تواند خطر شکستگی استخوان ناشی از پوکی استخوان را کاهش دهد. باید در نظر داشت که هیچ مرور سیستماتیکی نمی‌تواند جانشین یک مطالعه اولیه خوب طراحی شده شود؛ از این رو پیشنهاد می‌شود که مطالعات مشاهده‌ای آینده‌نگر با حجم نمونه کافی، گروه کنترل و مدت پیگیری مناسب طراحی و اجرا گردد.

Title: بررسی وضعیت ایمنی علیه کزاز در افراد بالای 50 سال در کاشان

http://salmandj.uswr.ac.ir/article-1-465-fa.html

اهداف: کزاز یک اختلال عصبی است که بصورت تک گیر وتقریباُ همیشه درافرادی که دارای مصونیت ناکامل می باشند بروز می کند. اکثریت موارد کزاز درافراد بالای 60 سال می باشد با توجه به خطر ابتلا به فرم شدید و مرگ و میربالای آن در بین افراد مسن این بیماری از اهمیت بالایی برخوردار است.این مطالعه به منظور بررسی سطح آنتی بادی ضد کزاز در افراد بالای 50 سال در شهرستان کاشان انجام گردید. مواد و روش‌ها: این مطالعه بصورت توصیفی درسال 1387 روی 180 فرد بالای 50 سال که به آزمایشگاه بوعلی کاشان برای انجام آزمایش چکاپ مراجعه کردند انجام شد. پس از کسب رضایت، پرسشنامه ای استاندارد حاوی اطلاعات دموگرافیک وسابقه واکسیناسیون کزاز تکمیل وسپس از هر فرد 5 سی‌سی خون گرفته و سطح آنتی بادی کزازبه روش الایزا اندازه گیری شد. نتایج بوسیله SPSS مورد تجزیه‌وتحلیل قرار گرفت و با آمار توصیفی ارائه گردید. یافته‌ها: %65 افراد ایمنی محافظت کننده علیه کزاز نداشتند (سطح آنتی توکسین کزاز کمتراز 0/1 IU/ml). از 180 فرد مورد مطالعه 72نفر(%40) سابقه دریافت واکسن نداشتند درحالی که 47 نفر (%26/1) حداقل یک نوبت واکسن دریافت کرده بودند.از میان 86 فرد بالای 60 سال 6نفر (%7) آنتی توکسین کزاز(1-0/1 IU/ml) و  5نفر (%5/8) آنتی توکسین کزاز (1≥IU/ml) داشتند.جنس مرد و دریافت قبلی واکسن با ایمنی محافظت‌کننده ارتباط داشت سطح آنتی توکسین با افزایش سن کاهش یافت. نتیجه‌گیری: به نظر می‌رسد اکثریت افراد بالای 50 سال دراین مطالعه به علت پوشش ناکافی واکسیناسیون نسبت به کزاز ایمن نمی‌باشند و نیاز به بهبود سطح ایمنی علیه کزاز دراین افراد وجود دارد.

Title: تاثیر مداخلات کاردرمانی مراجع مدار بر رضایتمندی و عملکرد سالمندان مبتلا به سکته مغزی در فعالیت‌های خودمراقبتی

http://salmandj.uswr.ac.ir/article-1-440-fa.html

اهداف: این پژوهش با هدف بررسی تاثیر مداخلات کاردرمانی مراجع مدار بر عملکرد ورضایتمندی افراد مبتلا به سکته مغزی در فعالیت‌های خودمراقبتی انجام شده است. مواد و روش‌ها: در این مطالعه 24 نفر از افراد سکته مغزی مراجعه‌کننده به مرکز توانبخشی رفیده به صورت نمونه در دسترس و با توجه به معیارهای ورود و خروج انتخاب و به صورت تصادفی در دو گروه کنترل و مداخله قرار گرفتند که 2 نفر از گروه مداخله از ادامه درمان خودداری کردند. سپس افراد در گروه مداخله 24 جلسه تحت مداخله کاردرمانی مراجع مدار در حیطه فعالیت‌های خودمراقبتی قرار گرفتند. مدت هر جلسه 45 دقیقه بود. گروه کنترل کاردرمانی رایج را دریافت کردند. رضایتمندی، عملکرد و استقلال در فعالیت‌های خود مراقبتی این افراد، قبل و بعد از مداخله ارزیابی شد. ابزارهای‌های جمع‌آوری اطلاعات شامل فرم مشخصات دموگرافیک، مقیاس ارزیابی عملکرد کاری کانادائی و آزمون بارتل بود. یافته‌ها: بین دو گروه در نمره رضایتمندی، عملکرد و استقلال در فعالیت‌های خودمراقبتی قبل از مداخله تفاوت معناداری وجود نداشت. بعد از مداخله هم بین دو گروه در نمره رضایتمندی، عملکرد و استقلال در فعالیت‌های خودمراقبتی تفاوت معناداری مشاهده تشد ولی قبل و بعد از مداخله درمانی در گروه مداخله اختلاف معناداری در نمره رضایتمندی (0/009=P)، عملکرد (0/028=P) و استقلال (0/019=P) در فعالیتهای خودمراقبتی مشاهده شد.همچنین اختلاف معنا داری در گروه کنترل بین قبل و بعد از مداخله در نمره رضایتمندی (0/011=P)، عملکرد (0/007=P) و استقلال (0/029=P) در فعالیت‌های خودمراقبتی مشاهده شد. نتیجه‌گیری: براساس نتایج فوق مداخله کاردرمانی مراجع مدار بر رضایتمندی، عملکرد و استقلال فعالیت‌های خود مراقبتی افراد مبتلا به سکته مغزی تاثیر داشته است اما این تاثیر آنقدر زیاد نبوده که این نتایج را بتوان در مقایسه دو گروه باهم مشاهده کرد، فقط این تاثیر در مقایسه بین قبل و بعد از مداخله در گروه مداخله مشاهده شد.

Title: افسردگی در سالمندان ترکمن - سال 1389

http://salmandj.uswr.ac.ir/article-1-441-fa.html

اهداف: روند افزایش جمعیت سالمند در دنیا به نحوی است که در سال 2050 بیش از %20 جمعیت جهان را سالمندان تشکیل خواهند داد که اکثریت آن‌ها در کشورهای در حال توسعه ساکن خواهند بود. اختلالات روحی روانی از جمله افسردگی از مهم‌ترین مشکلات سالمندان می باشد. از طرفی در گروه‌های قومی و اقلیت‌ها موانعی مانند زبان و تحصیلات پایین وجود دارد که می‌تواند منجر به اشکال در تشخیص اختلالات روحی روانی و شناختی گردد. لذا در این مطالعه مقطعی میزان شیوع افسردگی درسالمندان ترکمن بالای 60 سال مورد بررسی قرار گرفت. مواد و روش‌ها: مطالعه حاضر یک مطالعه توصیفی مقطعی می باشد که بر روی 300 سالمند ترکمن بدون اختلال شناخت و ساکن جامعه پس از اخذ رضایت شرکت در تحقیق انجام شد. نمونه‌گیری بصورت چند مرحله ای تصادفی خوشه‌ای بوده است. در هر شهرستان تعداد نمونه شهر و روستا بصورت تسهیم به نسبت جمعیت شهری و روستایی مشخص گردید. سپس با نمونه‌گیری خوشه‌ای تصادفی، خوشه‌ها تعیین شد. در این پژوهش از پرسشنامه بررسی افسردگی در سالمندان 15 (GDS) استفاده شد و به‌صورت مصاحبه با سالمند تکمیل گردید. یافته‌ها: بر اساس یافته های تحقیق میانگین نمره افسردگی در سالمندان ترکمن 3/353±4/31 بوده و میزان شیوع افسردگی خفیف 20 درصد، افسردگی متوسط 10 درصد، افسردگی شدید 3 درصد و67 درصد بدون علامت بودند. میانگین نمره افسردگی در زنان 3/193±5 و در مردان 3/380±3/66 و میانگین سن افراد مورد مطالعه در 7/06±69 سال و در زنان 6/48±67 سال بوده است. نتیجه‌گیری: افسردگی از مشکلات شایع سالمندان است، شیوع افسردگی متوسط و شدید در سالمندان ترکمن کمتر یا مشابه مطالعات دیگر در ایران می‌باشد. با توجه به شیوع افسردگی خفیف، لازم است برنامه ریزیهای لازم جهت شناسایی و درمان به موقع صورت گیرد. بررسی عوامل موثر بر افسردگی پیشنهاد می گردد.

Title: رابطه بین ویژگی‌های جمعیت شناختی و رضایت از بازنشستگی در سالمندان بازنشسته

http://salmandj.uswr.ac.ir/article-1-464-fa.html

اهداف: مطالعه حاضر با هدف بررسی رابطه بین ویژگی‌های جمعیت شناختی و رضایت از بازنشستگی در افراد سالمند عضو کانون بازنشستگی آموزش و پرورش شهر اهواز صورت گرفت. مواد و روش‌ها: این پژوهش از نوع توصیفی–همبستگی بود. در این مطالعه 96 سالمند با روش نمونه گیری در دسترس از مراجعان به کانون بازنشستگی آموزش و پرورش شهر اهواز در سال 1388 انتخاب شدند. برای سنجش میزان رضایت از بازنشستگی از آزمون شاخص توصیفی بازنشستگی (RDI) استفاده شد و به منظور بررسی ویژگی‌های جمعیت شناختی پرسشنامه‌ای توسط محققین فراهم گردید. جهت تجزیه و تحلیل داده‌ها از آمار توصیفی، همبستگی ساده و تحلیل رگرسیون با روش ورود مکرر استفاده گردید. یافته‌ها: بین سلامتی، اشتغال به کار مجدد پس از بازنشستگی، داشتن وضع مالی مناسب و احساس سودمندی با رضایت از بازنشستگی همبستگی مثبت معنی‌دار وجود دارد. رابطه بین سن بازنشستگی و بازنشستگی همسر با رضایت از بازنشستگی منفی است. نتیجه‌گیری: این پژوهش نشان می‌دهد که سلامتی، اشتغال به کار مجدد پس از بازنشستگی، داشتن وضع مالی مناسب و احساس سودمندی همگی رضایت از بازنشستگی را بالا می برند. اما سن بازنشستگی بالاتر و بازنشستگی همسر، رضایت از بازنشستگی را کاهش می‌دهد.

Title: مقایسه میزان شادکامی سالمندان ساکن در منزل و سرای سالمندان

http://salmandj.uswr.ac.ir/article-1-439-fa.html

اهداف: تحقیق حاضر با هدف مقایسه میزان شادکامی سالمندان ساکن در منزل و سرای سالمندان انجام شد. مواد و روش‌ها: این پژوهش از نوع تحقیقات علی‌ّمقایسه ای است. جامعه آماری این تحقیق تمامی زنان و مردان سالمند 60 سال به بالای ساکن در سرای سالمندان و ساکن در منزل شخصی در شهر تبریز بوده است که افراد نمونه 100 نفر در دو گروه 50 نفری (25 نفر مرد و 25 نفر زن) به روش نمونه گیری در دسترس انتخاب شدند. از سیاهه شادکامی آکسفورد استفاده شد و سپس داده ها با آزمون t مستقل بررسی شدند. یافتـه‌ها: نتایج نشان داد که میزان شادکامی سالمندان ساکن در منزل به طور معنی داری بیشتر از سالمندان ساکن در سرای سالمندان است. همچنین از میان مولفه های شادکامی، میزان عوامل رضایت از زندگی، سلامت روانی، خلق مثبت و کار آمدی سالمندان ساکن در منزل به طور معنی داری بالاتر از سالمندان ساکن در سرای سالمندان است. اما هر دو گروه در میزان عزت نفس تفاوت معنی داری نداشتند. نتیجه‌گیری: یافته های پژوهش حاکی از آنست که میزان شادکامی سالمندان ساکن در منزل به خاطر برخورداری آنها از حمایت اجتماعی و احترام و محبت خانواده به طور معنی داری بیشتر از سالمندان ساکن در سرای سالمندان است. و بالعکس سکونت در سرای سالمندان برای افرادی که می توانستند در کنار خانواده به سر ببرند اما به سبب شرایط اقتصادی و خانوادگی به سرای سالمندان سپرده شده اند، باعث کاهش خلق، عدم رضایت از زندگی و در نهایت عدم شادکامی شده است.

Title: مصرف داروی نامناسب در سالمندان ترخیص شده از بیمارستان‌های تحت پوشش دانشگاه علوم پزشکی تهران با استفاده از معیار Beers

http://salmandj.uswr.ac.ir/article-1-438-fa.html

اهداف: مطالعات نشان داده است که افراد مسن نسبت به دیگر گروه‌های سنی، بیشتر به بیماری‌های مزمن مبتلا شده و تحت درمان می‌باشند و در نتیجه از داروهای بیشتری استفاده می‌کنند. به دلایل بسیاری، سالمندان در معرض خطر مشکلات مرتبط با دارو هستند که از جمله آن‌ها تغییرات مرتبط با سن در فارماکوکینتیک (Pharmacokinetics) و فارماکودینامیک (Pharmacodynamics) داروها است که عملکرد داروها را در بیماران سالمند، تغییر می‌دهد. استفاده داروی نامناسب در بیماران سالمند، منجر به واکنش‌های دارویی نامطلوب می‌گردد. شناسایی این‌گونه داروها و عدم تجویز آنان برای این گروه سنی، مانع از بروز حوادث نامطلوب و ارتقای کیفیت خدمات درمانی برای آنان می‌شود. مواد و روش‌ها: مطالعه حاضر به صورت توصیفی-تحلیلی بر روی 212 نفر از سالمندانی که در رده سنی 60 سال و بالاتر قرار داشتند و در دو بیمارستان عمومی- آموزشی و دو بیمارستان عمومی-غیرآموزشی تحت پوشش دانشگاه علوم پزشکی تهران بستری و سپس ترخیص شده بودند، انجام گرفت. پرسشنامه معیار Beers (Beers criteria) به منظور شناسایی مصرف داروهای نامناسب مورد استفاده سالمندان و تداخلات دارو – بیماری در سالمندان 60 سال و بالاتر، به عنوان ابزار سنجش مورد استفاده قرار گرفت. یافته‌ها: میانگین سنی سالمندان مورد مطالعه، 69/32(6/82=sd) سال بود. مصرف داروی نامناسب در میان سالمندان %20/75 بود. بیشترین داروهای نامناسبِ مورد استفاده، آلپارازولام (%16/66)، کلوردیازپوکسید (14/28%)، فوکستین (11/90%) و اُکازپام (11/90%) برآورد گردید و بیشترین میزان تداخل دارو-بیماری مربوط به تداخل ناهنجاری های لخته خون با داروی کلوپیدگرل ‏(29/4%) بود. بیشترین طبقه دارویی مورد استفاده سالمندان مورد مطالعه از داروهای نامناسب، بنزودیازپین‌ها (%49/98) بودند. رابطه آماری معنی‌داری بین میزان درآمد با مصرف داروی نامناسب وجود داشت (0/041=P). نتیجه‌گیری: میزان مصرف داروی نامناسب در میان سالمندان در مقایسه با بسیاری از کشورها زیاد می‌باشد. مصرف چنین داروهایی، سالمندان را در معرض واکنش‌های دارویی نامطلوب قرار می‌دهد. نتایج این مطالعه نشان می‌دهد که نیاز به تدوین و عملیاتی نمودن دستورالعمل‌های علمی استفاده از داروها، ارتقای معیارهای استفاده از آنها، و بهبود آگاهی پزشکان وجود دارد و این امر می‌تواند منجر به پایش توام با دقت در رژیم دارویی بیماران سالمند می‌گردد. برگزاری دوره‌های آموزشی برای پزشکان برای آموزش چنین مواردی ضروری به نظر می‌رسد.
